# Patient experiences of acute medical same day emergency care: a semi-structured interview study across two hospitals in England

**DOI:** 10.1136/bmjopen-2026-125048

**Published:** 2026-11-06

**Authors:** Sue Dean, Julian Barratt, Jo Leonardi-Bee, Holly Blake

**Affiliations:** 1 Same Day Emergency Care, United Lincolnshire Teaching Hospitals NHS Trust, Boston, UK; 2 School of Health Sciences, University of Nottingham, Nottingham, UK; 3 School of Psychology, Aston University, Birmingham, UK; 4 Centre for Evidence Based Healthcare, University of Nottingham, Nottingham, UK; 5 NIHR Biomedical Research Centre: Nottingham, Nottingham, UK

**Keywords:** Emergency Service, Hospital, GENERAL MEDICINE (see Internal Medicine), HEALTH SERVICES ADMINISTRATION & MANAGEMENT, Patient-Centered Care, Patient Satisfaction, QUALITATIVE RESEARCH

## Abstract

**Abstract:**

**Objectives:**

Same day emergency care (SDEC) has expanded across England to reduce admissions and improve patient flow, yet evidence on patient experience remains limited. This study explored patients’ experiences of acute medical SDEC and identified factors shaping positive and negative perceptions.

**Design:**

Qualitative study using semi-structured interviews and reflexive thematic analysis.

**Setting:**

Two SDEC units within a single National Health Service Trust in England.

**Participants:**

16 adults who attended acute medical SDEC. Participants were recruited during attendance and interviewed shortly afterwards by telephone or via Microsoft Teams.

**Methods:**

Interviews explored communication, waiting, staff interactions, involvement, privacy, symptom management, the environment, transitions and overall experience. Data were analysed inductively using reflexive thematic analysis, supported by regular discussions with academic supervisors and a patient representative to enhance credibility.

**Results:**

Five themes captured participants’ experiences of SDEC as a responsive, safe and well-organised environment. Clear explanations, regular updates and opportunities to ask questions supported understanding and reassurance. The calm atmosphere, timely assessment and relational qualities of care, empathy, attentiveness and respectful interactions, were central to positive experience. Participants felt able to express preferences and participate in decisions to the extent they wished. Challenges arose mainly at service boundaries, particularly during transitions from other services, information transfer and uncertainty about referral pathways. These system-level issues shaped the wider journey but did not diminish confidence in the care received within SDEC.

**Conclusions:**

SDEC was experienced as a positive and person-centred model of urgent care, with communication, relational interactions and timely processes central to satisfaction. Challenges at service boundaries highlight the importance of coordinated pathways and clear information during transitions. This study provides new insight into patient experience of SDEC and offers learning relevant to international efforts to develop ambulatory emergency care models that deliver timely, coordinated and person-centred treatment outside traditional inpatient pathways.

**Trial registration details:**

https://doi.org/10.17605/OSF.IO/2CB6K

**STRENGTHS AND LIMITATIONS OF THIS STUDY**

- Use of semi-structured interviews enabled rich, detailed accounts of communication, relational care and system navigation.
- Data were collected across two same day emergency care sites within the same National Health Service Trust, allowing exploration of experiences in more than one service setting, although findings may still reflect organisational practices specific to this Trust.
- Recruitment through clinical teams may have introduced selection or courtesy bias.
- Conducting interviews virtually or by telephone may have limited access to non-verbal cues and affected the depth of some accounts.
- The predominantly older, white British sample, together with the absence of staff perspectives and observational data, limited the breadth and triangulation of the findings.

## Introduction

 Although same day emergency care (SDEC) builds on earlier ambulatory care models, implementation in England has been largely policy-driven, with services introduced at pace across a wide range of specialties and with considerable variation in local delivery.^1 2^ Similar, although differently configured, models have been implemented internationally, despite a limited evidence base.^3^

National bodies view SDEC as central to the transformation and future sustainability of urgent and emergency care (UEC), particularly in the context of rising demand, constrained inpatient capacity and an ageing population.^4–6^ Acute medical patients represent a substantial proportion of emergency admissions to hospitals, yet many are discharged within 24 hours, suggesting that same-day pathways can safely meet this need while reducing pressure on hospital beds.^1 7^ These ambitions align with wider system priorities set out in the National Health Service (NHS) Fit for the Future: 10-Year Health Plan for England.^8^

Alongside these operational goals, patient experience is recognised as a fundamental dimension of healthcare quality.^9–12^ Evidence consistently links positive patient experience with safer care, better clinical outcomes and more effective use of services, including improved adherence and reduced avoidable utilisation. This body of work positions patient experience within national frameworks not as a peripheral or ‘soft’ aspect of care, but as a core marker of quality.^13–15^

National frameworks reinforce this perspective. The National Institute for Health and Care Excellence quality standard for patient experience emphasises dignity, respect, involvement in decisions, clear communication and coordinated care as essential components of high-quality services and is supported by further guidance.^16 17^ NHS England’s Patient Experience Book highlights links between positive experience, improved outcomes and lower costs.^18^ Patient experience is central to the Care Quality Commission’s regulatory strategy,^10^ and embedded within the NHS Outcomes Framework.^9^

Despite the rapid expansion of SDEC and the centrality of patient experience to quality, recent reviews highlight a striking absence of published research exploring how patients experience this model of care.^19 20^ Understanding patient experience is particularly important in SDEC, where patients often encounter a fast-paced environment, multiple staff interactions and rapid clinical decision-making. Qualitative inquiry can help illustrate how patients make sense of this model of care, what aspects of the service shape their experiences and where improvements may be needed to ensure high-quality and equitable care.

This study therefore sought to explore, in depth, how patients experienced acute medical SDEC in two hospitals within a large rural and coastal NHS Trust in Lincolnshire. The county has dispersed communities, pockets of socioeconomic deprivation and longstanding challenges in delivering timely urgent and emergency care. Within this context, SDEC provides a structured pathway for ambulatory medical patients who require rapid assessment and treatment, supports same-day decision-making, reduces unnecessary admission and helps maintain flow across acute medical services, including offering an alternative route for some patients who might otherwise remain in the emergency department (ED) for assessment. Through qualitative interviews, the study aimed to identify what mattered most to patients, what supported or hindered positive experiences and how SDEC services might be strengthened from the patient perspective.

## Methods

### Study design

This exploratory qualitative interview study used semi-structured interviews and reflexive thematic analysis to explore patient experiences of acute medical SDEC. Semi-structured interviewing provided a balance between consistency across participants and flexibility to elicit individual perspectives. The study was informed by a broadly pragmatic qualitative approach, recognising that patient experiences are shaped by the wider organisational and healthcare contexts in which care is delivered.

The interview guide (online supplemental file 1) was finalised prior to data collection in line with ethics requirements and was used as a prompt rather than a script, allowing participants to raise any aspect of their SDEC experience. The study’s patient and public involvement and engagement (PPIE) representative reviewed it to ensure clarity, relevance and acceptability. Reporting was guided by the Consolidated Criteria for Reporting Qualitative Research (COREQ).^21^

### Setting

The study took place in two acute medical SDECs within a single NHS Trust, which are open for 14 hours a day, 7 days a week. The SDECs are staffed by a combination of advanced practitioners and medics, along with nurses, healthcare assistants, ward clerks and housekeepers. SDEC provides rapid assessment and treatment for patients referred from primary care, the ED and ambulance services, with the aim of delivering timely diagnostics and management while avoiding unnecessary admission. Patients are typically seen for a focused episode of care that involves clinical review, investigations and short periods of waiting as results are processed. The pace is brisk and the environment is oriented toward throughput, which shapes how patients move through the service and how they experience their contact with staff. This context is central to interpreting the accounts shared in the interviews.

### Sampling and recruitment

Purposive sampling was used to recruit patients who had attended acute medical SDEC within the previous month, ensuring that their experiences were recent enough to support reliable recall. Clinical staff in each unit identified eligible patients during their visit and briefly introduced the study; patients could choose to opt in. Participation was voluntary, individuals who declined were not asked to give reasons and no record was maintained of the number of individuals who declined. Patients who expressed interest were contacted by the researcher after discharge to discuss participation, receive written information about the research team and purpose of the study and provide written informed consent. Sampling aimed to include variation in age, sex, gender identity, disability and ethnicity.

A provisional sample of 24 participants was planned to capture a range of patient experiences across the SDEC pathway and provide sufficient breadth of perspectives across differing patient characteristics and care experiences. However, recruitment concluded after 16 interviews. During data collection, the researcher reviewed each interview and compared participants’ accounts with those already collected. Later interviews largely repeated previously identified experiences and did not add substantively different perspectives relevant to the study aim. Sample sufficiency was therefore guided by the concept of information power, which emphasises the relevance and richness of data in relation to the study aim rather than numerical saturation.^22^

### Data collection

Semi-structured interviews were conducted between November 2024 and May 2025 by a clinical academic PhD student researcher (SD) trained in qualitative interviewing and supervised by academics with expertise in qualitative methods. Participants chose online (Microsoft Teams) or telephone interviews; none opted for an in-person format. Remote interviewing can limit access to non-verbal cues but may also enhance comfort and openness and did not appear to compromise rapport or depth.^23 24^

The researcher had no prior relationship with participants. Interviews were conducted one-to-one, except for one participant who requested their spouse be present. Interviews lasted 12–30 min, were audio-recorded with consent and transcribed verbatim. No repeat interviews were conducted. Field notes were written immediately after each interview to capture contextual details, early analytical impressions and reflexive observations.^25 26^ All data were stored securely on institutional systems.

### Data analysis

Data were analysed using reflexive thematic analysis.^26^ SD read transcripts repeatedly and carried out inductive coding across the full dataset. In line with reflexive thematic analysis, coding was undertaken by a single researcher, with rigour supported through sustained engagement with the data and analytical discussion rather than multiple-coder agreement. Codes were then grouped to identify early patterns, and potential themes were generated and developed iteratively by moving between coded extracts, full transcripts and the researcher’s developing interpretations. Themes were refined for coherence and distinctiveness and shaped into a final thematic structure. Because participant numbers per site were small and demographic variation limited, analysis was conducted at the aggregate level. Theme development was supported through regular discussion within the supervisory team and with a PPIE representative. NVivo (V.14) was used to organise and manage data.

Interview transcripts were not returned to participants, consistent with critiques that transcript review may conflate accuracy with validity and place unnecessary burden on participants.^27–29^ Informal member checking took place during interviews through real-time summarisation and clarification to confirm the researcher’s understanding of participants’ accounts. Participants were not asked to review the completed analysis or findings.

### Reflexivity

The doctoral researcher (SD) is a white British female trained in mixed methods and qualitative interviewing; she conducted all interviews and initial coding. SD is employed as an advanced practitioner within emergency medicine and previously worked clinically in the ED, but at the time of the study worked in acute medical SDEC. This insider position supported rapport, contextual understanding and clinically informed probing, but also risked assumptions about shared understanding, privileging clinically familiar issues or influencing the interpretation of participants’ comparisons between SDEC and the ED. An outside researcher might have provided greater critical distance and challenged assumptions arising from familiarity with both settings, although they may have had less understanding of the clinical context.

SD was mindful of the power dynamic inherent in a clinician-researcher interviewing patient participants and sought to reduce this through open, non-directive questioning and by emphasising participants’ expertise in their own experiences.

Reflexive field notes were kept throughout data collection and analysis, capturing assumptions, emerging questions and moments of uncertainty. These notes helped contextualise the transcripts, supported reflexive awareness of the researcher’s influence and contributed to the development of early analytical insights. They also informed regular discussions with the supervisory team and a PPIE representative, whose challenges and alternative interpretations strengthened the credibility and grounding of the final themes.

### Trustworthiness

Trustworthiness was considered through credibility, transferability, dependability and confirmability. Credibility was supported through sustained engagement with the data, real-time summarisation and clarification during interviews, analytical discussion within the research team and PPIE involvement in interpreting developing themes. Detailed descriptions of the participants, service settings and study context were provided to enable readers to assess transferability. Dependability was supported through transparent documentation of data collection and the iterative analytical process. Confirmability was supported through reflexive field notes, explicit consideration of the researcher’s positionality and analytical discussions that examined assumptions and developing interpretations.

### Patient and public involvement

Patients and members of the public contributed to the design of the study, including refinement of the protocol and review of all participant information materials. A PPIE representative also supported data interpretation by reviewing and discussing developing themes. Patients were not involved in data collection.

### Ethical approval and consent

Data were managed in accordance with NHS information governance requirements, including secure storage and removal of identifiable information from transcripts. Participants were reminded that involvement was voluntary and that they could withdraw at any time without consequence. No participants reported distress during interviews, and no safeguarding concerns were identified.

The Health Research Authority advised that naming participating sites was a decision for the host organisation and the university as study sponsor. Following consultation, both agreed that publicly naming the research sites in Lincolnshire would provide useful contextual information; the sites had already been identified in the published study protocol.^30^ No additional participant consent was required for site naming.

### Findings

16 patients who had recently attended SDEC services participated in semi-structured interviews. Participants reflected on their experiences across the SDEC care pathway, offering insight into communication, relational aspects of care, operational processes and perceptions of clinical quality.

### Participant characteristics

Participant demographics are summarised in table 1. All identified as white British. Participants were evenly distributed across the two SDEC sites.

**Table 1:** Participant characteristics

Characteristic	Participants (n=16)
Age: mean (SD), years	63 (15)
Gender, n (%)	
Female	10 (62.5)
Male	6 (37.5)

Note: *All participants identified as male or female. All participants were white British.

### Overview of themes

The analysis generated five themes that illustrate how participants experienced SDEC services across the two hospital sites (table 2). While participants were invited to reflect on both positive and negative aspects of care, accounts were predominantly positive. Nonetheless, some identified areas for improvement, particularly relating to transitions between services and the clarity of information during waits.

**Table 2:** Themes and subthemes

Theme	Subthemes
Communication and sense making	Understanding the purpose of SDEC. Clarity of explanations and next steps. How information shaped confidence.
Relational care and emotional safety	Empathy and reassurance. Feeling listened to and respected. Privacy and emotional comfort.
Involvement and autonomy	Opportunities to ask questions. Participation in decisions. Feeling able to influence care.
System navigation and boundaries	Waiting and flow through the service. Responsiveness to needs. Understanding what SDEC can and cannot provide.
Perceived quality and safety	Thoroughness of assessment. Confidence in clinical decision-making. Judging safety through staff behaviour and processes.

SDEC, same day emergency care.

### Narrative presentation of themes

The following sections present each theme, with supporting quotations that illustrate how participants described their experiences of SDEC.

#### Theme 1: communication and sense-making

Communication shaped how participants understood and navigated their SDEC visit. Clear explanations about symptoms, diagnoses and clinical decisions helped people make sense of what was happening and reduced uncertainty:

She explained everything and was very thorough…talked through everything. (P3)

One of the consultants…explained what would happen if the results were this way or that way. (P7)

Some appreciated that communication extended to family members when needed:

One of the doctors actually rang my sister…told her about my diagnosis…that was really good. (P14)

In contrast, unclear or inconsistent communication, usually referred to by participants who had attended the ED prior to SDEC, created confusion:

I kept getting asked why I was there…no-one seemed to have read the letter. (P11)

Participants valued transparency about processes and delays, particularly when staff explained why waits occurred:

We seem to be waiting a long time for the results and she said…at this time of night…there’s no doctors to look at the results in this hospital…somebody somewhere else in the world will look at them…that gave me reassurance… because they…inform you what’s going on. (P12)

When updates were absent, participants were unsure whether they were waiting for tests or had been overlooked:

I’ve been sat here 40 minutes…am I waiting on some tests or has something happened? (P11)

Understanding next steps was central to feeling prepared for discharge. Most felt well informed about follow-up plans, self-management and who to contact if their condition changed:

I’ve got to go for further tests on my heart…It’s a very good system this SDEC. (P1

They explained what I needed to do at home and who to call if things change which was very helpful. (P2)

I was given a clear plan of what to do if I was unwell…If you’re concerned, come back to us. (P6)

A minority described more limited information about onward care:

Just the discharge letter…that they would write to the doctor. (P13)

This differed from accounts of clear safety-netting and follow-up planning. For this participant, the discharge information appeared primarily administrative and provided limited guidance about onward care or what to expect next.

These accounts suggest that communication in SDEC was shaped not only by interpersonal skill but also by participants’ perceptions of the structure of the service. Participants contrasted SDEC’s smaller scale and more predictable workflow with previous experiences of ED, where they perceived that high demand limited staff availability. They felt that these differences created greater opportunities for staff to provide clear explanations and consistent updates. Participants’ positive evaluations were often filtered through expectations formed in ED, where long waits and limited information were familiar. This expectation framing helps explain why communication in SDEC was experienced as particularly strong.

#### Theme 2: relational care and emotional safety

Participants consistently described SDEC as a warm, reassuring environment where staff were approachable, attentive and emotionally supportive. These relational qualities helped participants feel safe and cared for during a period of uncertainty.

Staff who took time to talk, answer questions or offer calm reassurance were particularly valued:

He gave me some life tips…very professional…gave you a good sense you were being looked after. (P10)

They actually do make eye contact there. (P12)

Small gestures, including a cup of tea, a check-in, a moment of kindness, were remembered as meaningful:

When you’re stressed…a cup of tea can go down really well…staff were just friendly but professional. (P4)

Participants contrasted this with their previous experiences of ED, where they perceived that busyness limited emotional support. SDEC felt calmer, more personal and more relational:

It just felt like a cosy corner of the hospital where you knew everybody. (P5)

Privacy and dignity were also part of emotional safety. Most felt their confidentiality was protected through curtains, lowered voices or private rooms:

The curtains were pulled round…I had no concerns about my privacy. (P3)

One participant noted that layout occasionally compromised privacy. Although this differed from the predominant accounts, it appeared to reflect the physical configuration of the environment rather than staff conduct.

Relational, person-centred, care appeared to be a defining feature of participants’ experiences of SDEC. Staff visibility and continuity of contact seemed to create a microculture where emotional support was possible in ways participants did not associate with their previous experiences of ED. At the same time, the overwhelmingly positive tone of accounts may reflect a degree of gratitude bias, where patients soften critique because they feel relieved, cared for or are aware of wider system pressures. Emotional safety, in this sense, appeared to be co-produced by both organisational conditions and patient expectations.

#### Theme 3: involvement and autonomy

Participants valued being able to express preferences, ask questions and participate in decisions about their care. Feeling listened to contributed to a sense of partnership and respect.

Some described confidently declining tests or negotiating aspects of care:

They came and…said we’ll have an ECG [electrocardiogram]. I said, I’ve only just had one…I don’t see any need for another one…I’ve had two this morning, so that was it. I didn’t have one. (P1)

This participant exercised autonomy actively by questioning the proposed investigation and declining its repetition.

Others appreciated staff anticipating their needs, such as breastfeeding support:

I didn’t have to fight…they were already thinking about how to support that. (P5)

Here, involvement was experienced not through having to request or negotiate support, but through staff recognising the participant’s needs in advance.

Most felt included in decision-making:

I was informed, I was given the opportunity to ask any questions. (P6)

For some participants, involvement meant being informed and invited to ask questions rather than directing clinical decisions.

A few preferred a more hands-off role, trusting staff expertise:

The decision was obviously theirs…I was satisfied with what I was getting. (P15)

This differed from more active accounts of involvement but did not necessarily indicate exclusion from decision-making. Instead, it reflected the participant’s preference to defer to professional expertise while remaining satisfied with the care received.

Although participants described feeling able to express preferences, their accounts also indicate subtle power dynamics. Opportunities for involvement were usually initiated by staff, through offering options, inviting questions or explaining decisions, rather than participants actively directing care. One participant explicitly deferred to professional expertise, but others described trusting clinicians’ judgement and feeling comfortable with staff leading decisions. This pattern indicates that autonomy in SDEC is shaped by both staff behaviours and participants’ own assumptions about their role in urgent care, rather than reflecting shared decision-making in its fuller sense. The absence of negative accounts may reflect a tendency to moderate criticism, with participants emphasising positive interactions rather than challenging clinical authority.

#### Theme 4: system navigation and boundaries

Participants’ experiences were shaped by how they moved through the SDEC pathway, including waiting times, sequencing of tests and the clarity of instructions. Many described the service as efficient and well-coordinated:

Everything seemed to run very smoothly…I never felt like I was just sitting around waiting. (P12)

Responsiveness, from clinical and non-clinical staff, reinforced this sense of flow:

They keep you informed all the time…checking on you if you need anything. (P12)

However, uncertainty sometimes arose when instructions were unclear or when participants were unsure what they were waiting for:

I sat there for about 40 minutes not knowing what was happening. (P11)

Transitions into SDEC could involve repetition of information or unclear boundaries between services. One participant described being directed to ED by their GP rather than referred directly to SDEC, followed by a prolonged wait during which information did not appear to transfer effectively:

I then went to A&E and was there for about 17 hours…kept getting asked why I was there; no-one seemed to have read the letter. I had to keep answering the same questions. (P11)

Some participants reflected on wider system pathways, suggesting they could have been directed to SDEC earlier by the general practitioner or ED:

The only thing that would have been better is if I’d gone there straight away rather than sat in A&E [Accident & Emergency]. (P8)

Another participant questioned whether healthcare professionals were sufficiently aware of SDEC services and referral opportunities:

Sometimes I think health professionals are a little bit hesitant to refer to SDEC…not quite sure they’re fully aware of all the services you do offer…And so I do wonder if your services are used enough by professionals. (P6)

These experiences highlighted the tension between SDEC’s intended efficiency and the realities of wider system pressures. Uncertainty often arose at points where SDEC intersected with primary care, ED or diagnostic services, suggesting that participants did not always experience transitions or information transfer between services as seamless. Some perceived that greater professional awareness of SDEC might have enabled more direct access, although referral routes were not systematically explored across the sample. Participants’ willingness to tolerate delays or confusion appeared to be shaped by comparisons with their previous ED experiences, reinforcing the role of expectation framing in how SDEC is evaluated. These accounts suggest that patient experience was shaped not only by interpersonal care within SDEC but also by referral pathways, patient flow and coordination across service boundaries.

#### Theme 5: perceived quality and safety

Participants generally expressed strong confidence in the clinical care they received. They consistently described feeling that symptoms were taken seriously, assessments were thorough and decisions were explained, which contributed to perceptions of safety:

They listened to my symptoms, gave me pain relief…sorted out my fit note…a very efficient, friendly service. (P6)

For this participant, quality was experienced through the combination of symptom relief, practical support and an efficient process, rather than through clinical treatment alone.

Timely action for more complex needs reinforced this:

They arranged blood tests and a transfusion the same day…they understood the urgency. (P11)

The speed with which investigations and treatment were arranged signalled that the participant’s condition had been recognised and taken seriously. Timeliness therefore contributed not only to efficiency but also to reassurance and perceived safety.

Seeking second opinions or repeating checks was interpreted as thoroughness:

He was really worried it was a stroke, so he got another doctor. (P12)

Involving another clinician was not interpreted as uncertainty or lack of competence. Instead, it reassured the participant that the concern was being treated seriously and that staff were prepared to seek confirmation before reaching a clinical decision.

Most concerns were addressed promptly when raised:

The cannula was feeling funny…so they took it out and put a new one in. (P14)

However, one participant described being asked to apply pressure to a cannula site without her pre-existing weakness being recognised:

She told me to put pressure on it, but I have weakness on my right side from a stroke years ago…my hand kept slipping…I felt that that was still bleeding and when I got home I took off my jacket, my arm, my jumper were saturated in blood. (P11)

This experience differed from the predominant accounts of responsive and individualised care. Although it did not negate the participant’s wider account of SDEC, it demonstrated how failure to consider functional limitations when giving discharge instructions could create avoidable risk after leaving the service.

Cleanliness and visible attention to detail also contributed to perceptions of safety:

It was spotless…they were there wiping all the chairs down. (P9)

Cleanliness provided another observable indication that care was organised and that risks were being managed.

Perceptions of safety were closely tied to visible markers of thoroughness, including timely treatment, repeated checks, second opinions and cleanliness. Participants interpreted safety through observable behaviours rather than through technical details of clinical care. The less positive account showed that perceived safety also depended on whether instructions were adapted to the individual’s functional abilities and remained workable after discharge.

While concerns were often minimised or contextualised, they provided contrasts to the predominantly positive accounts. This may reflect trust in staff, relief at avoiding admission or awareness of pressures elsewhere in the system. Perceived quality therefore appeared to be constructed through clinical processes, visible staff behaviours and the relational environment.

### Overall reflections

Across themes, participants described SDEC as a positive and reassuring model of care, combining efficient clinical processes with strong relational support. Suggestions for improvement focused mainly on the physical environment and wider system pathways, such as earlier referral to SDEC, clearer information transfer and smoother transitions, rather than on the service itself. Their accounts suggest that positive experiences of SDEC are shaped not only by interpersonal interactions but also by participants’ perceptions of the structural and cultural features of the model.

Preferred levels of involvement varied. Some participants actively negotiated aspects of care, while others valued being informed and were comfortable with clinicians leading decisions. Perceptions of quality and safety were similarly shaped by visible indicators of thoroughness and responsiveness, including timely treatment, repeated checks and second opinions. However, an isolated account in which instructions did not account for a participant’s functional limitation demonstrated the importance of adapting care to individual abilities and circumstances.

Participants frequently contrasted SDEC with previous encounters in the ED, and these comparisons appeared to influence how they framed their accounts, often highlighting SDEC’s calmer pace, clearer communication and more predictable flow. These subjective comparisons do not establish that SDEC provides better care than ED, as the study was not designed to compare the two services and they operate with different functions, patient populations and system pressures. However, this expectation framing appeared to shape how participants understood and evaluated their SDEC experiences. Experiences before reaching SDEC also indicated that referral decisions, professional awareness of available pathways and coordination between primary care, ED and SDEC could influence the wider care journey.

Gratitude for timely care and awareness of system pressures may also have influenced how concerns were expressed, with critical reflections perhaps delivered in understated ways. Overall, the analysis indicates that patient experience of SDEC was shaped through the interaction of relational care, organisational processes, individualised support, preferred forms of involvement and expectations formed through previous urgent care encounters.

## Discussion

This study explored patients’ experiences of SDEC and found that participants generally viewed the service as responsive, supportive and clinically safe. Clear communication, emotional reassurance and timely care were central to how participants understood and navigated their experience. Although some challenges were noted, often relating to earlier stages of the urgent care journey rather than SDEC itself, the overall picture was one of confidence and satisfaction. These findings address the study aim by illustrating what patients value within SDEC and by highlighting the interpersonal and organisational features that contribute to positive experience. As one of the first qualitative studies to examine SDEC from the patient perspective, this work provides insight into how urgent care redesign is experienced on the ground and what relational and organisational components appear to matter most.

The importance participants placed on communication aligns with wider UEC research, which consistently identifies clear explanations, regular updates and opportunities to ask questions, as key contributors to reassurance and trust.^31 32^ Studies of ED settings frequently report that limited information and long waits can heighten anxiety and reduce perceptions of care quality.^33 34^ In contrast, the SDEC environment described by participants as more orderly, more predictable and more communicative, appears to support a different kind of patient experience. However, these subjective comparisons should not be interpreted as evidence that SDEC provides better care, as the study was not designed to compare the two services and did not account for their different functions, patient populations or operating conditions, including the substantial system pressures affecting ED care.

Timeliness and efficiency were also important. Participants valued prompt assessment but emphasised that waiting was experienced more positively when accompanied by transparency and acknowledgement, echoing findings from studies showing that communication shapes how patients interpret delays.^35–38^ Staff visibility and responsiveness have similarly been shown to mitigate the emotional impact of waiting in emergency settings,^39 40^ consistent with participants’ accounts here.

Relational aspects of care such as kindness, attentiveness, respect and opportunities for involvement, were central to person-centred care and positive experiences.^41^ This aligns with the wider literature emphasising the value of relational care in UEC, even though it can be difficult to sustain in pressured environments.^42–44^ Relational care provides a useful concept for understanding these findings because it positions attentiveness, connection and recognition of the patient as integral to care rather than additional interpersonal qualities. In participants’ accounts, staff visibility, continuity of contact and small acts of kindness appeared to reduce uncertainty and create emotional safety. These interactions supported trust in the service and, in some cases, confidence in the clinical care itself. Participants’ accounts suggest that SDEC may offer more space for relational interaction than traditional ED pathways, supporting findings that smaller, well-coordinated, urgent care services can feel more personal and easier to navigate.

The findings suggest that SDEC offers a model of urgent care in which interpersonal, organisational and clinical elements, combine to create a supportive environment. Structural features, such as smaller scale, clearer workflow and a calmer atmosphere, appear to enable the relational attentiveness and communication that participants valued. In this sense, SDEC may function as a ‘buffer zone’ within a pressured system, absorbing demand that would otherwise fall to ED and creating conditions more conducive to personalised care. These mechanisms resonate with wider debates on urgent care transformation, where workforce configuration, patient flow models and integrated pathways are seen as central to improving patient experience.^45–47^

Although accounts were predominantly positive, several factors may have shaped how participants framed their experiences. Recruitment via clinical teams and telephone or virtual interviews may have introduced social desirability or courtesy bias, particularly within an NHS context where patients may feel reluctant to criticise staff.^48–50^

Participants frequently compared SDEC favourably with ED, suggesting that expectations shaped by previous experiences of crowded or delayed emergency care may have amplified perceptions of SDEC as efficient and reassuring. These dynamics indicate that positive accounts should be interpreted in relation to both the service itself and the broader system pressures against which it was evaluated. These accounts represent participants’ perceptions rather than a direct evaluation of the two services and do not establish that SDEC is superior to ED. Nevertheless, the consistency of this perceived contrast warrants further investigation.

Transitions into and out of SDEC were another point where experience varied. Several participants described delays or uncertainty during their preceding ED attendance, consistent with evidence that early-journey waiting and pathway ambiguity can heighten anxiety.^51 52^ Some reported uncertainty about referral pathways, including perceptions that primary care services were not always aware of direct routes into SDEC, while other accounts highlighted repetition and poor information transfer between services. As referral routes were not systematically explored across the sample, these findings cannot establish how consistently direct access pathways were understood or used. However, they indicate that patient experience may depend partly on coordination between general practice, ED and SDEC. A small number also described uncertainty during discharge, reflecting wider challenges in emergency care transitions.^53–55^ These findings suggest that while SDEC itself was experienced positively, wider system processes can shape the overall journey.

### Implications for practice and service development

The findings point to several areas with practical relevance:

- Communication and updates were central to patient confidence, particularly during waiting periods or transitions.
- Relational care, attentiveness, respect and emotional support were consistently valued and appear integral to a positive person-centred experience.
- Referral pathways could be improved, as some participants felt they could have been directed to SDEC earlier, echoing concerns about pathway complexity in UEC.^56–58^
- Discharge processes may benefit from clearer information.

These insights suggest that the strengths of SDEC lie not only in rapid assessment but also in the relational and organisational conditions that support a more person-centred experience.

### Limitations

The study’s trustworthiness was considered through credibility, transferability, dependability and confirmability, as described in the Methods.

This study has several limitations. Data were collected across two SDEC sites within a single NHS Trust serving a rural and coastal county with socioeconomic challenges. Although the detailed description of the setting supports assessment of transferability, the findings may reflect local organisational practices and patient populations and may not transfer directly to SDEC services operating in different contexts.

Although efforts were made to recruit a varied sample, participants were predominantly older and white British, limiting insight into the experiences of younger adults and people from minority ethnic backgrounds. These groups are often under-represented in research participation and may report different care experiences.^59^ As a result, the findings may reflect the expectations and communication norms of this demographic group rather than universal experience. Cultural factors, perceptions of authority and differing levels of confidence in navigating urgent care may shape how other groups experience SDEC, including potential inequities in access or involvement.

Non-response bias is also possible, as individuals who chose to participate may differ from those who declined; younger adults, people living in deprived areas and minority ethnic groups are known to be less likely to engage in research of this kind.^60^ Recruitment was facilitated by clinical teams, and information about patients who declined an initial invitation was not available to the research team; potential non-participation bias could therefore not be assessed.

Interviews were conducted shortly after attendance, supporting recall but potentially influenced by the emotional intensity of urgent care encounters. As with all interview studies, accounts reflect participants’ interpretations rather than direct observation of clinical interactions. Finally, the study focused solely on patient perspectives; including staff views or observational data may have provided additional insight into how SDEC processes operate in practice.

### Future research

Future research could explore how SDEC is experienced across a wider range of organisational contexts, including services with different staffing models, patient populations and levels of demand. Studies involving younger adults and people from minority ethnic backgrounds are particularly needed, given their under-representation in this study and in urgent care research more broadly. Including staff perspectives or observational methods may also provide additional insight into how communication, relational care and workflow processes operate in practice. Finally, further work examining referral pathways and transitions into SDEC could help address the system-level issues that shaped some participants’ experiences and support more consistent access to SDEC.

## Conclusion

This study provides new insight into how patients experience SDEC and illustrates the features that shape a positive, person-centred, urgent care journey. Participants consistently valued clear communication, emotional reassurance, respectful interactions and timely processes, describing SDEC as a supportive and clinically safe environment. Preferred levels of involvement varied, ranging from actively negotiating aspects of care to being informed while allowing clinicians to lead decisions. Experiences of quality and safety also depended on whether care and instructions were responsive to individual needs and abilities. Challenges were most evident at the boundaries of the service, particularly during transitions from primary care or ED, information transfer between services and discharge communication, emphasising the need for coordinated pathways and reliable information flow.

By centring the patient perspective, this study strengthens the limited evidence base on SDEC and offers learning relevant to international efforts to redesign urgent and acute care. Many health systems are seeking alternatives to traditional inpatient admission and overcrowded EDs, and comparable models, such as ambulatory care units, rapid assessment clinics and short-stay medical services, are increasingly being adopted. Although these models differ in their organisation and function, the findings highlight the relational and organisational elements that underpin positive experiences within these models, including staff visibility, attentive communication, individualised support and a calm, predictable environment.

Improving referral pathways, information transfer, discharge communication and the adaptation of care to individual needs, while ensuring equitable access to same-day services may further strengthen patient experience and support the wider shift toward ambulatory emergency care internationally. As health systems continue to redesign urgent and acute care, understanding how patients navigate and interpret these emerging models offers practical direction for service development, quality improvement and global efforts to deliver timely, person-centred care.

## Supplementary material

10.1136/bmjopen-2026-125048 online supplemental file 1

## Data Availability

No data are available.
