# Supplementary material for: Patient experiences of acute medical same day emergency care: a semi-structured interview study across two hospitals in England
Source: BMJ Open. 2026 Nov 6;16(11):e125048. doi: 10.1136/bmjopen-2026-125048 (PMC13756465; doi:10.1136/bmjopen-2026-125048)
Supplement: Supplementary file 2 [file bmjopen-16-11-s001.docx]

**Semi-structured Interview Schedule – The SDEC Study**

To be conducted either face to face on hospital premises, or remotely via Teams, depending on the participant’s preference, date and time to be agreed with the participant. Written consent to be obtained.

**Opening:** Welcome and ensure the participant is comfortable. Confirm understanding of the purpose of the study: to discuss experiences of SDEC including key themes (arrival, waiting, staff, care and treatment, tests, pain, environment and facilities, leaving), aiming to influence policy to benefit future patients. Confirm consent if given prior to the interview, and remind participant that the interview will be recorded. Anticipated length of interview 30 minutes.

**Main Content:**

| Themes | Potential questions |
| --- | --- |
| Arrival | Who referred you?  Mode of transport?  Reception? |
| Waiting | How long did you wait to see a nurse or doctor?  Information given on waiting times/reasons?  Length of visit?  Was the visit length as you expected? |
| Staff | Enough time to discuss your issue with clinicians?  Were you listened to?  Were explanations understandable?  Were any anxieties and fears discussed?  Did you have confidence in the staff? |
| Care and treatment | Were you given enough information about your condition or treatment?  Was privacy/dignity maintained?  Did you get help from staff if you needed it?  Were you given conflicting information by staff?  Were you as involved as you wanted to be in decisions? |
| Tests | If you had tests, were these explained, including the reason?  Were the results explained or were you told how you would get the results if they were not available? |
| Pain | If you had pain, did staff help to resolve this? |
| Environment and facilities | Was the department clean?  Did you feel safe?  Could you access food and drink if allowed? |
| Leaving SDEC | Did you go home?  Were any new medications explained?  Were potential side effects explained?  Were you advised on resuming usual activities?  Did staff ask about your home situation or support?  Were you advised when to seek further medical attention and/or who to contact if you had concerns? |
| Demographics | Gender  Age  Ethnicity  Disability  Hospital site |

**Closing:**

Summarise the main points that were discussed; brief update on next steps: no further input is required from the participant, data will be transcribed and used for thematic analysis, anonymity will be maintained, findings will be written up and disseminated. If the participant would like to receive information about the findings of the research, their name and contact details should be provided to facilitate this. Thanks for participation.
